# Cervical Cystic Teratoma: An Unusual Cause of Airway Obstruction in the Pediatric Population

**DOI:** 10.7759/cureus.19166

**Published:** 2021-10-31

**Authors:** Huzaifa Azam, Muhammad Amir Hanif, Muhammad Imran Khan, Ayousha Masood, Muhammad Usman Hashmi

**Affiliations:** 1 Anesthesiology, The Children's Hospital and The Institute of Child Health, Multan, PAK; 2 Pediatric Surgery, The Children's Hospital and The Institute of Child Health, Multan, PAK; 3 Anaesthesiology, Chaudhry Pervaiz Elahi Institute of Cardiology, Multan, PAK; 4 Internal Medicine, Faisalabad Medical University Hospital, Faisalabad, PAK; 5 Internal Medicine, Nishtar Medical University Hospital, Multan, PAK

**Keywords:** airway obstruction, non-seminomatous germ cell tumors, cervical cystic teratoma, case report, extragonadal germ cell tumor, infants, anterior neck mass, mature cystic teratoma

## Abstract

Cystic teratoma is a germ cell tumor, which usually involves the gonads. However, it can be located occasionally in other organs. The most common extragonadal sites for germ cell tumors include midline structures such as the retroperitoneum, mediastinum, pineal body, and supra-sellar space. Here, we describe a case of a patient who presented with a cystic teratoma involving the anterior aspect of the neck. The chief complaints of the patient consisted of a large swelling in front of the neck, difficulty in breathing, and frequent regurgitation of milk during feeding. Initially, a diagnosis of cystic hygroma was instituted for which the patient underwent sclerotherapy utilizing bleomycin. However, no improvement was observed in the patient’s condition. A detailed evaluation was planned, starting with a CT scan of the head and neck that suggested cystic teratoma as the likely etiology. Surgical excision of the mass was performed, and an excisional biopsy for histopathological examination was taken. A final diagnosis of cervical cystic teratoma was established based on the histopathological findings. The purpose of reporting this case is to raise awareness among fellow healthcare professionals that cystic teratoma can also present with a large swelling in the anterior neck with obstructive features.

## Introduction

Cystic teratoma is a type of germ cell tumor that consists of cells from all three germ layers i.e. endoderm, mesoderm, and ectoderm. A review of the available medical literature reveals that these tumors often contain hair, teeth, cartilage, glands, and bones. Owing to the presence of various embryological derivatives many theoretical mechanisms have been put forward to explain its pathogenesis. These include aberrant fertilization, abnormal meiotic division causing spontaneous asexual development of an unfertilized ovum, or variability of blastodermal elements in a fertilized ovum. However, the exact etiology is still unclear and warrants further research [1-3].

We can classify cystic teratoma into three broad categories, namely monodermal teratoma, mature cystic teratoma, and immature cystic teratoma, out of which mature cystic teratoma is the most commonly encountered clinical entity. Mature cystic teratoma is a slow-growing benign tumor. However, these tumors can rarely be transformed into a malignant variant. The prevalence of this malignant transformation is estimated at 1% to 2% of the cases [4]. As far as the site of involvement is concerned, the ovaries and testes are the two most common sites for the development of cystic teratoma. Rarely these tumors can also be present in extragonadal sites including the sacrococcygeal region, anterior mediastinum, retroperitoneal space, pineal body, and above the sella turcica [5]. Moreover, cervical cystic teratomas are seldom reported in the medical literature. These cervical cystic teratomas can extend towards the thoracic cavity and cause airway obstruction by local mass effect, leading to breathing difficulties in such patients [6]. The purpose of reporting this case is to raise awareness among fellow healthcare professionals that cystic teratoma can also present with a large swelling in the anterior neck with obstructive features.

## Case presentation

A 10-month-old male patient presented with a large swelling in front of the neck associated with shortness of breath and stridor for two weeks which was worsening over time. The mother also reported regurgitation of milk from the patient’s nostrils and difficulty in feeding. The parents noticed this swelling soon after birth, prompting them to seek medical advice. The primary care physician diagnosed the swelling as a case of cystic hygroma based on the clinical features. Hence, the patient underwent two sessions of sclerotherapy with bleomycin, four months apart. However, no improvement was seen. So the primary care physician referred him to a tertiary care hospital for further evaluation and management.

**Figure 1:**
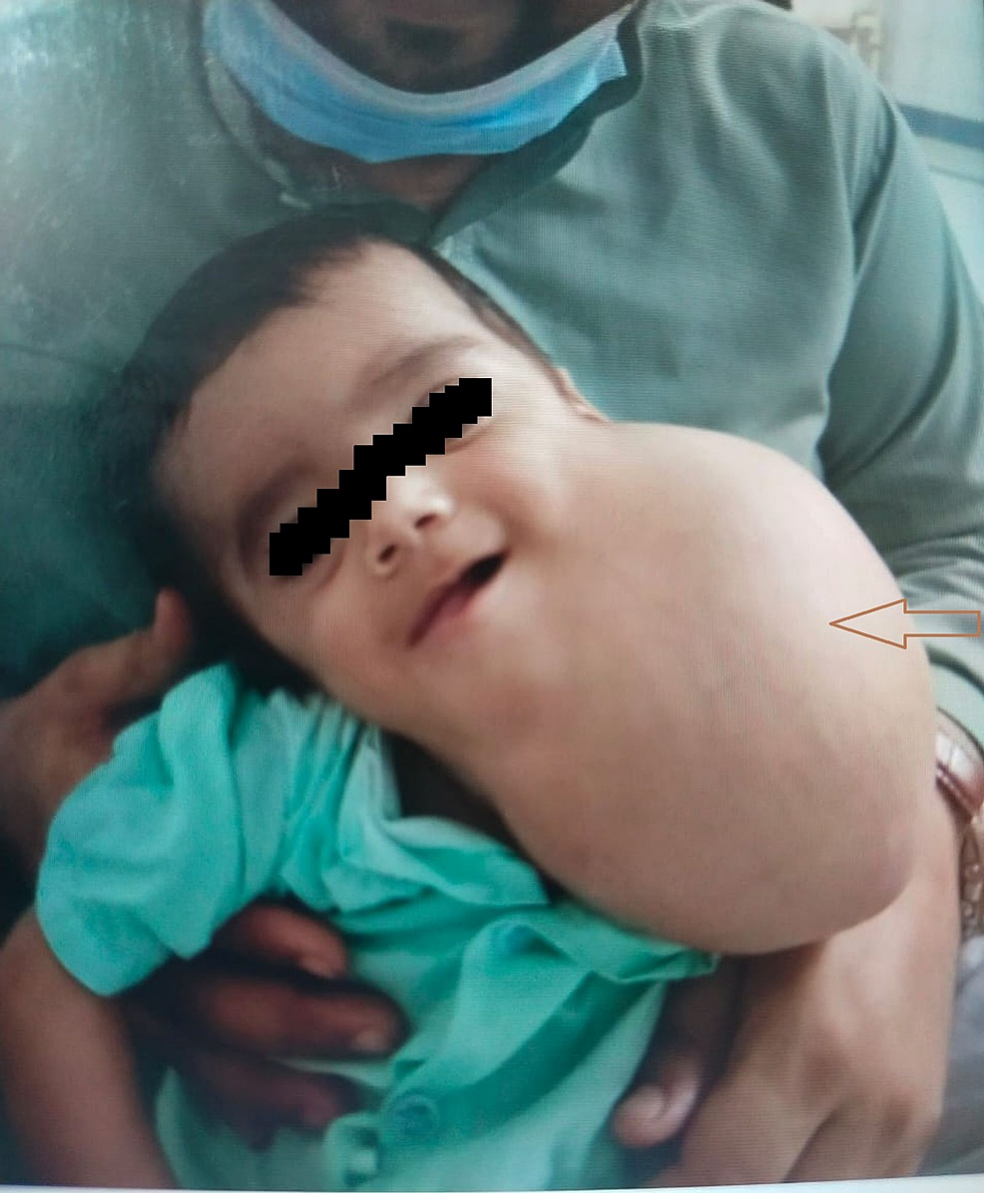
A large anterior neck swelling with overlying erythema as marked by the arrow. It extends from the left temporomandibular joint to the clavicle and encroaches towards the angle of the right mandible.

In the tertiary care hospital, the patient was re-evaluated through a detailed history and thorough general physical examination, which revealed a fluctuant cystic anterior neck swelling with overlying erythematous skin. It extended from the left temporomandibular joint to the clavicle and encroached towards the angle of the right mandible. Figure 1 depicts these findings.

**Figure 2:**
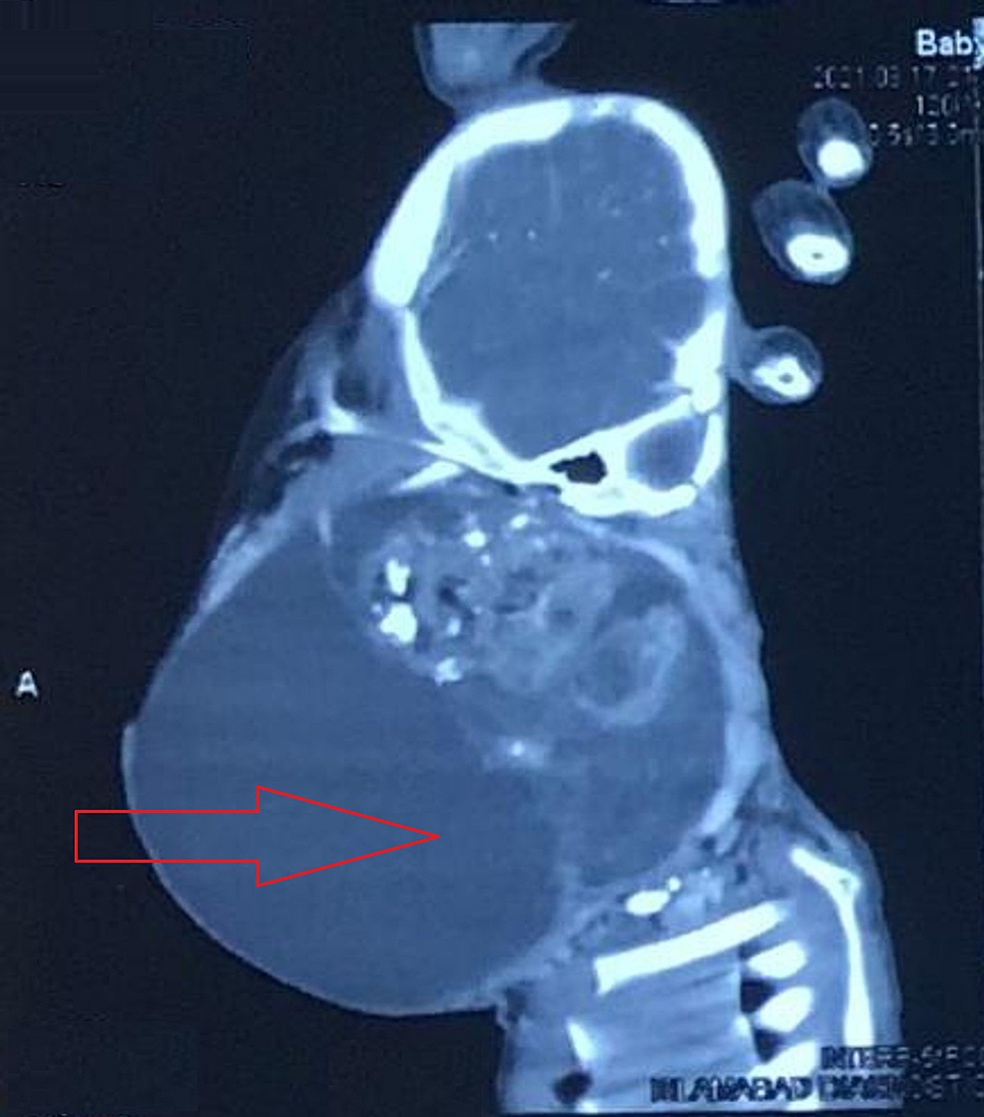
The CT scan of the head and neck shows a large exophytic mass with solid and cystic components on the left side of the neck as marked by the arrow. The mass is compressing adjacent structures.

On palpation, the mass was multilobulated with mixed cystic and solid components and no regional lymphadenopathy. Auscultation revealed wheeze and crackles on both sides of the lung fields. During the preoperative workup, a CT scan of the head and neck was performed which showed a large exophytic mass with solid and cystic components on the left side of the neck. This mass was compressing adjacent structures including the mandible, pharynx, larynx, upper trachea, and esophagus. Figure 2 shows these findings. 

These imaging findings were more suggestive of cervical cystic teratoma as compared to cystic hygroma. Hence, after completion of the preoperative workup, the patient was planned for elective surgery to excise the mass. The surgery was complicated by a one-centimeter transverse breach in the cervical esophageal wall which was primarily repaired. Postoperative recovery was uneventful. Histopathology of the excisional biopsy revealed a lesion comprising of cells from all three germ layers. Unfortunately, on the seventh postoperative day, the patient developed aspiration pneumonitis during the process of nasogastric feeding. Therefore we shifted the patient to pressure-controlled ventilation. After one week, he started to maintain oxygen saturation, so he was shifted back to the pediatric surgery floor after being weaned off the ventilator. He was closely monitored for three days. His condition remained stable, so he was discharged with instructions to follow up after one week. Table 1 describes the timeline of all these events.

**Table 1:** Timeline for the case report showing the chronology of the various events. CBC: Complete blood count; CT scan: Computed tomography scan; ICU: Intensive care unit; AFP: Alpha-fetoprotein.

Dates	Summaries of initial and follow-up visits	Diagnostic testing	Interventions
22-10-2020	Initial visit at a primary healthcare physician clinic with complaints of difficulty in breathing and frequent regurgitation of milk during feeding. On examination, there was a large-sized anterior neck swelling. The primary care physician established an initial diagnosis of cystic hygroma.	Baseline laboratory investigations that were within the normal reference range	Sclerotherapy with bleomycin
19-02-2021	No improvement in symptoms		Repeated sclerotherapy with bleomycin
16-06-2021	The swelling increased in size despite two attempts of sclerotherapy		The patient was referred to the tertiary care hospital for further evaluation.
18-06-2021	The patient was re-evaluated by detailed clinical examination and imaging studies. Based on clinico-radiological findings, the patient was diagnosed with cervical cystic teratoma. Surgery was planned after optimization of the patient.	CBC showed low hemoglobin and hematocrit. Chest X-ray revealed tracheal deviation to the right side and a mass in the left upper chest. A CT scan of the head and neck showed a large exophytic mass with solid and cystic components on the left side of the neck	The patient’s clinical condition was optimized. He received antibiotics, steroids, nebulization, and chest physiotherapy.
22-06-2021	The patient underwent surgical excision of the mass and had an uneventful recovery	The excisional biopsy was sent for histopathology.	Routine ICU care
25-06-2021	Naso-gastric feeding was started. Histopathology report confirmed the diagnosis of mature cervical teratoma		Routine ICU care
30-06-2021	On the seventh postoperative day, the patient developed severe shortness of breath, excessive drooling, and coughing. He was not able to maintain oxygen saturation. Aspiration pneumonitis was considered as a likely etiology.	Chest x-ray revealed bilateral infiltrates. Blood cultures and sputum cultures came out to be negative.	We shifted the patient to pressure-controlled ventilation. He also received nebulization and chest physiotherapy. The patient was kept nil per oral and received Intravenous fluids.
10-07-2021	The patient started to maintain oxygen saturation, so he was shifted back to the pediatric surgery floor after being weaned off the ventilator. He was closely monitored for three days. His condition remained stable, so he was discharged with instructions to follow up after one week.		
17-07-2021	The 1st follow-up visit after surgery: The patient was asymptomatic.	Serum AFP was within the normal reference range.	
20-08-2021	The 2nd follow-up visit after surgery: The patient remained asymptomatic with no recurrence.	Serum AFP was within the normal reference range.	

## Discussion

Cervical cystic teratoma is a rare type of germ cell tumor seen in the pediatric population [7]. Owing to the atypical location of a cervical cystic teratoma, differentiation from other cervical masses is quite difficult. Therefore, these masses tend to remain misdiagnosed or have delayed diagnosis. Similarly, our patient was initially misdiagnosed as a case of cystic hygroma for which he underwent sclerotherapy, which led to a delay in exact diagnosis and initiation of optimal curative therapy. Furthermore, this resulted in unnecessary interventions and an undue economic burden.

Compressive features are frequently observed in patients with a large cystic teratoma due to mass effects on the adjacent structures causing airway obstruction, dysphagia, and dyspnea. In cases of airway obstruction, early treatment is mandatory as failure to timely intervene can result in an 80% to 100% mortality rate [8]. Interestingly, our patient also had airway obstruction causing shortness of breath. The process of intubation was difficult, and we observed tracheal shifting.

This case was diagnosed in the postnatal period and could not be diagnosed in the antenatal period. This can be attributed to the lack of appropriate antenatal screening scans. During a review of the literature, we found a similar case where a patient was misdiagnosed as a case of cystic hygroma, and sclerotherapy was performed as a primary intervention, but no response to therapy pointed towards an alternative diagnosis [9]. 

The process of establishing a diagnosis of cervical teratoma is quite a difficult task. Imaging studies play a crucial role in the diagnostic work-up. Therefore, ultrasonography is an efficient, convenient, and safe modality to diagnose these cases both in the prenatal and postnatal periods. The characteristic features of cervical teratoma on ultrasound include a mass with cystic and solid components which is usually multiloculated and has multiple septa. Half of the cases show dispersed calcified areas [10]. A CT scan is also helpful in differentiating various differentials of cervical cystic teratoma. It is worth a mention that the CT scan findings of our case were consistent with the classic findings described in previous research studies [11,12]. Moreover, the MRI scan has also proven its significance as it provides valuable information regarding local invasion, anatomy, and relation to surrounding structures. All these features help a physician gather accurate information to plan a surgery [10,12]. In this case, we did not need an MRI, as the radiological findings of the CT scan and CT angiogram were conclusive for the establishment of diagnosis.

In addition to imaging studies, serum tumor markers and histopathology of excised tissue are recommended. Alpha-fetoprotein (AFP) is a tumor marker that plays a role both in the diagnosis and evaluation of germ cell tumors [13]. In this case, the preoperative and postoperative AFP levels were in the normal reference ranges. This finding is consistent with a previous research study in which AFP levels were normal in 95.8% of cases of mature cystic teratoma. In contrast, malignant cystic teratomas presented with elevated AFP levels [14]. 

The management of cervical cystic teratoma is complex and requires a multidisciplinary approach. Therefore, an expert and well-trained team of pediatricians, pediatric surgeons, pulmonologists, and anesthesiologists should participate in the management of these cases. This team approach can help to reduce the risk of complications. Surgical excision of the mass is the standard treatment that is curative in most cases [6].

In our patient, a postoperative complication of aspiration pneumonitis developed during the seventh postoperative day. We suggest this might be due to a hasty initiation of nasogastric feed. This factor delayed the recovery period of the patient and prolonged the intensive care unit stay. Therefore, the authors suggest that nasogastric feed should be started while adopting precautionary measures. These precautionary measures include the upright position of the patient, elevating the patient's head end by 30 degrees, avoiding nasogastric tube feeding before going to sleep, and elevating the head of the patient after feeding [15]. In this way, we can improve the surgical outcome by preventing or decreasing the frequency of chemical pneumonitis during the postoperative period.

## Conclusions

Cystic teratoma is a germ cell tumor that can also involve extragonadal sites. Cervical cystic teratoma is an exceedingly rare tumor of the pediatric population that can present with a large anterior neck mass compressing the adjacent structures. If a child presents with a neck swelling associated with features of airway obstruction, dysphagia, and dyspnea, cervical cystic teratoma should also be included in the differentials. The healthcare physician should explore these cases with the help of detailed imaging studies for early diagnosis and prevention of potentially fatal complications.
